# Development of a Diet Production System for *Conopomorpha cramerella* (Lepidoptera: Gracillariidae), a Major Cocoa Production Pest in Southeast Asia and the Pacific Islands

**DOI:** 10.3390/insects14080708

**Published:** 2023-08-14

**Authors:** Jerome Niogret, Anisah Binti Savantil, Arni Ekayanti, Mavis Peter Jaus, Wulan Wulan, Elviah Mitzo, Jean-Philippe Marelli, Desmond Conlong

**Affiliations:** 1 Mars Wrigley, Nguma-bada Campus, James Cook University, Smithfield, QLD 4878, Australia; 2 Centre for Tropical Environmental & Sustainability Science, Nguma-bada Campus, James Cook University, Smithfield, QLD 4878, Australia; 3 Malaysian Cocoa Board, Pusat Penyelidikan Bioteknology Koko, Kota Kinabalu 89200, Sabah, Malaysia; anisah@koko.gov.my (A.B.S.); mavis@koko.gov.my (M.P.J.); elviah@koko.gov.my (E.M.); 4 Mars Cocoa Research Centre, Mars Wrigley, Tarengge, Luwu Timur 92971, Sulawesi Selatan, Indonesia; arni.ekayanti@effem.com (A.E.); wulan.wulan@effem.com (W.W.); 5 Mars Wrigley, Plant Sciences Laboratory, Davis, CA 95616, USA; jean-philippe.marelli@effem.com; 6 Department of Conservation Ecology and Entomology, Stellenbosch University, Private Bag X1, Matieland 7602, Stellenbosch, South Africa; desconlong.bugs@gmail.com

**Keywords:** artificial diet, cocoa pod borer, *Conopomorpha*, laboratory rearing system, pest, cacao, development

## Abstract

**Simple Summary:**

This study focused on developing an artificial diet for rearing the cocoa pod borer, a pest insect that affects cocoa plants. The researchers compared the success rates of two diets, MM1 and MM4, jointly developed by Mars Wrigley and the Malaysian Cocoa Board. They analyzed the nutritional composition of the diets and their effects on the overall development and success rates of the cocoa pod borer. The modifications made to the MM1 diet resulted in the latest MM4 version. The results showed significant improvements in all stages of the cocoa pod borer development, including increased egg hatching rates, larval development, pupation success rates, and adult emergence. The duration of larval and pupal development was also reduced. The findings suggest that the MM4 diet could be a viable method for laboratory rearing of the cocoa pod borer. This research contributes to the understanding of insect diet development and provides practical implications for pest management strategies in the field.

**Abstract:**

The development of artificial diets for the cocoa pod borer *Conopomorpha cramerella*, a major pest of cocoa plants, has undergone significant advancements. In this study, we present the success rates of two diet formulations, MM1 and MM4, which have been progressively improved. Nutritional composition analysis revealed that the MM1 diet differed from the natural host, cocoa pods, in several aspects, including protein, carbohydrate, and vitamin C content. To address these differences, modifications were made to the diet compositions, leading to the MM4 diet version. These modifications resulted in improved diet quality and reduced contamination, leading to enhanced success rates in all stages of *C. cramerella* development. Larval development, pupation success rates, and adult emergence rates were significantly higher in the MM4 diet compared with the MM1 diet. Moreover, the duration of larval development and pupal stage decreased, while adult longevity increased with the MM4 diet. The overall development success of diet-reared insects from egg to adult was comparable with that of insects reared on cocoa pods. However, the cocoon formation, body length and fresh weight of the adults reared on the artificial diets were lower than those reared on cocoa pods. This diet formulation provides a promising approach for laboratory rearing of *C. cramerella* and opens avenues for further research and mass-rearing initiatives to mitigate the impact of this pest on cocoa production.

## 1. Introduction

Chocolate is a world favourite. The global chocolate market value reached over USD 130 billion globally in 2019 [1]. Despite cocoa being in the world’s top 10 traded commodities, 38% of the crop is lost to pests and diseases. In Southeast Asia and the Pacific regions, a widely distributed and indigenous micro-lepidopteran, the cocoa pod borer *Conopomorpha cramerella* (Snellen) (Lepidoptera: Gracillariidae), is considered the main threat to cocoa production in Indonesia, the Philippines, Malaysia, and Papua New Guinea [2].

*Conopomorpha cramerella* is polyphagous, with a number of host plants in the families Sapindaceae and Malvaceae [3]. Rambutan (*Nephelium lappaceum*) is considered the primary host of *C. cramerella*, and cocoa is considered a secondary host [4,5]. On cacao, female *C. cramerella* lay their eggs on the surface of the host pod. Upon hatching, the neonate larva bores directly through the cocoa pod epidermis to reach the endocarp and pulp (mucilage) where it feeds, disturbing the beans’ development and their nutrient supply [6,7]. The bean development disruption causes bean clumping and pod hardening, resulting in harvesting and processing difficulties. In addition, larval tunneling causes premature pod yellowing and hardening, which often results in premature harvesting and thus reduced bean quality [8]. Once fully developed, the late instar larvae exit the pod through its epidermis and produce a silk thread to lower themselves to a suitable pupation site, such as leaves on the host plant, fallen leaf litter, leaves of plants in the undergrowth, and/or the host fruit surface. The final larval instar larvae spin a cocoon and pupate after approximately 3–4 days. Adult emergence occurs after another 6–8 days [8].

This insect causes varying yield losses, from 20% to complete crop loss [8,9,10], leading to substantial economic losses estimated at around USD 500 million per year in Asia in the early 2000s [2]. Attempts to control the pest have been costly and ineffective, contributing to significant drops in cocoa production in Malaysia and Indonesia [1,11,12]. Various methods have been tried to manage the pest, including frequent harvesting and insecticide use, but successful control has been elusive due to its cryptic nature. To develop an effective integrated pest management (IPM) program, understanding the pest’s life cycle, biology, and behavior is crucial.

An essential tool in the IPM approach is to have a mass-rearing system in place under controlled environmental conditions and defined artificial diets so that biological parameters can be determined; additionally, insect material is necessary for host plant resistance studies, as hosts for biological control agents, and for field studies on the insects’ physiology, dispersal and behavior [13]. Marelli et al. [14] further state that new and emerging technologies need to be investigated for the control of *C. cramerella.* One such control tactic, which is ideally suited as a component of IPM, is the sterile insect technique (SIT) or its derivative, the concept of F1 sterility, which has been shown to be very effective for certain species of Lepidoptera [15]. The key component of SIT is a professionally run mass-rearing facility, incorporating an irradiator [13].

Despite the importance of having a good laboratory colony available, the published literature on artificial diet development for *C. cramerella* and other species in the Gracillariidae is sparse. The most promising diet developed (based on sustaining the larvae for only 11 days as the trial subsequently ceased due to high contamination) was initiated by Malaysian Cocoa Board researchers in the late 1990s [16]. Santoso et al. [17] had similar attempts in Indonesia. Their diets were based on general Lepidoptera diets used without much success on *C. cramerella* by previous researchers. Unfortunately, due mostly to diet contamination, diet development has continued to be unsuccessful. Awang et al. [18] composed 50 different meridic artificial diets for *C. cramerella*. The best formulation enabled them to successfully rear a colony from fertile eggs to moths (only at a 1% success rate) but this could not be sustained. They acknowledged that their diet quality and contamination issues were of concern and needed further research.

Since 2019, Mars Inc. and the Malaysian Cocoa Board have driven a concerted effort to improve artificial diets to provide a consistent supply of high-quality *C. cramerella* so that an effective IPM program can be developed against it. This work was conducted in parallel at the Mars field station in Tarengge, Sulawesi, Indonesia, and at the Malaysian Cocoa Board Biotechnology Centre in Kota Kinabalu, Sabah, Malaysia. In this paper, we describe the development of a diet production system from the Mars Cocoa Research Center in Indonesia by comparing the success rates and duration of each development stage of the pest life cycle. The development of an artificial diet for *C. cramerella* would significantly help further cocoa research programs in Southeast Asia, including but not limited to ecological and behavioral studies, the development of monitoring and mitigation, and a first step towards a potential SIT program.

## 2. Materials and Methods

### 2.1. Insect Collection and Laboratory Site Descriptions

The Entomology laboratory is part of the Mars Cocoa Research Center in Tarengge (MCRC), Sulawesi, Indonesia (2°33′20″ S; 120°47′56″ E). The research station consists of a 30 ha cocoa plantation where Mars Inc. conducts various research on cocoa agronomy, genetics, and pest and disease management.

To obtain field-collected pupae, *C. cramerella*-infested cocoa husks were placed on the ground and covered with dry cocoa leaves. Once leaving the cocoa husks, larvae seek a place to spin a cocoon and pupate underneath leaf material. A day later, the undersides of the dry leaves were checked for cocoons. When cocoons were found, the piece of leaf on which they were formed was cut out and brought back to the laboratory. The leaf pieces were placed, cocoon-side upward, into Bug-Dorm cages (Knitted mesh polyester; W60 × D60 × H60 cm, Australian Entomological Supplies PTY LTD, South Murwillumbah, NSW, Australia) until adult emergence. Upon emergence, the adults were sexed, as described in [9], and placed in another Bug-Dorm. The moths were able to feed and drink through a cotton wick emerging from plastic containers containing 10% honey solution or water.

### 2.2. Conopomorpha Cramerella Laboratory Rearing and Diet Development

#### 2.2.1. Eggs Collection

Immature cocoa pods (clone M01, D ± 7.5 cm and L ± 14 cm) were washed using paraben-free natural antibacterial liquid soap (Willow ark extract, Sukabumi, Indonesia), rinsed with sterile distilled water and sprayed with Ethanol 70% (OneMed, Sidoarjo, Indonesia). Once dried, the cacao pods were hung by the stem 15 cm high at the center of the rearing insect Bug-Dorm. The pods were then lightly sprayed with sterile distilled water before being covered by Kimwipe sheets (Kimwipes Kimtech Science, Roswell, NM, USA). Ten pairs of freshly emerged male and female *C. cramerella* were transferred to the Bug-Dorm overnight for mating and oviposition. A 3 cm piece of lamp wick (Richmond dental wick, Rockingham, NC, USA) was soaked with a 10% honey solution (Madu Murni Nusantara, Solo, Indonesia) and placed in a 3 cm plastic petri dish, without its lid, inside the bug-dorm. The females laid their eggs singly on the Kimwipe-covered surface of the cocoa pod overnight, 24 h after introduction. The small laid eggs, which were visible due to their orange oval shape, were counted under a stereoscopic microscope (Integrated CMOS microscope camera Leica IC90 E, Leica, Singapore). The freshly laid eggs were collected by cutting out a square (5 × 5 mm) of Kimwipe containing an egg. After cutting, 200–250 such egg papers were placed in a 10 cm glass petri-dish, covered with the lid, and kept at ambient conditions in the diet preparation room for 2 days. The chorion of *C. cramerella* eggs is fragile, especially during the first two days. Consequently, only 2-day-old eggs were used for the following decontamination process.

#### 2.2.2. Egg Decontamination

Because of potential microbial contamination of the Kimwipe oviposition substrate and/or the egg, the 2-day-old eggs were decontaminated before being transferred into the diet. The eggs attached to the Kimwipe squares were washed in a solution of 15 mL of 75% ethanol and 10 mL of 0.2% clorox in 75 mL of sterile distilled water, made up daily. The volume used to decontaminate batches of eggs was based on using 2 mL of the decontamination solution per egg. A screwcap 250 mL glass Erlenmeyer flask (Iwaki Co., Ltd., Bangkok, Thailand) was used to wash the eggs in the decontaminating solution by gently handshaking them for 5 min. The eggs were separated from the decontaminating solution by slowly pouring the solution from the flask. They were rinsed with clean distilled water for 3 min (2 mL of the sterile distilled water per egg). The eggs were then filtered from the distilled water wash using a porcelain Büchner funnel (Lab Logistics Group Labware, Meckenheim, Germany, diameter 7 cm) coated with sterile Kimwipe. The Kimwipe and eggs were allowed to dry on a running laminar flow bench (Heraguard Eco Thermo Scientific, Langenselbold, Germany). Once dried, the Kimwipe squares and their attached eggs were transferred using forceps (Dumont 1151-20 Knox No. 5, Dumont SA, Montignez, Switzerland) directly onto the diet surface, eggs facing up.

### 2.3. Artificial Diet Materials and Methods

#### 2.3.1. Diets Evaluated

The original Know-How on the diet recipe developed by the Malaysia Cocoa Board was used as the reference diet (MM1), against which changes in composition and methodology were compared as diets were further jointly developed. Over the development period, a number of changes were made to diet MM1 to make it closer to its cocoa pod natural host. This culminated in diet MM4, which is currently used. The formulation of both diets is shown in Table 1.

#### 2.3.2. MM1

The ingredients were categorized into groups A–G based on their types, with group A including the fresh ingredients, group B the dry ingredients, group C the vitamins and minerals, group D the lipids, group E the gelling agent, and group F and G including the ascorbic acid and antimicrobial blends, respectively.

The dry ingredients (Group B: cellulose powder, lecithin granules, tapioca flour, and brewer’s yeast) were evenly mixed by hand using a chemical stainless steel spoon (vol. 18/10 mL, Dumont, Inox, Swiss) in a 500 mL stainless steel beaker (Bochem Instrumente GmbH, Weilburg, Germany) and covered with aluminum foil.

Both vitamins (Group C: Myo-inositol and choline chloride) were dissolved in 20 mL of sterile reverse osmosis (RO) water in a 100 mL screwcap Erlenmeyer flask.

Ascorbic acid was dissolved separately in a 500 mL glass beaker (Iwaki Co. Ltd., Bangkok, Thailand) containing 100 mL of RO water.

Phytagel (Group E) was weighed into a 1000 mL Erlenmeyer flask (Iwaki, Thailand) which had been filled with 380 mL of sterile RO water and immediately hand-shaken until there were no lumps.

This gelling media (phytagel and RO water) and the dry ingredients were autoclaved (high-pressure steam sterilizer FLS-1000, NV-L 22AMU, Tomy Digital Biology Co., Ltd., Tokyo, Japan) at 115 °C for 20 min. Immediately after autoclaving, the gelling agent media was placed on a hotplate stirrer (IKA C-MAG HS 7, temp. 100 °C and Mot.1) to keep the phytagel at boiling point and the dry ingredients (Group B) were placed under laminar flow bench.

The surfaces of the locally sourced fresh plum tomatoes (Tomoni Fresh market, East Luwu, Indonesia) and chicken eggs (Group A) were washed with natural antibacterial liquid soap (Sleek baby, Bottle, Nipple, and Accessories cleanser, Active ingredients: Sodium lauryl sulfate 3%; Aqua, Sodium lactate, Sodium gluconate, Sodium benzoate 1%; Propanane-1,3-diol, Willow ark extract) before being sterilized in a 0.2% Clorox (Bayclin, Indonesia) solution for 15 min, rinsed using sterile distilled water, and dried out under a laminar flow bench (Binder climatic chamber KBF 240, Tuttlingen, Germany).

Following the surface wash, the fresh tomatoes were dipped in boiling water for 20 s to loosen the skin, which eased peeling. The seeds were removed before dicing the tomato into 3 × 3 mm pieces. The weighed diced tomatoes were added to a 2000 mL glass beaker containing 100 mL of the ascorbic acid solution, and the raw egg yolk, after being carefully separated from the egg white using an egg yolk separator (Krischef, Jakarta, Indonesia) and weighed, was added, and the mixture was stirred until homogeneous using a spatula. The diced tomato and egg yolk mixture was microwaved (Panasonic NN-ST342, Shanghai, China) on the high setting for 2 min, taken out and stirred, then microwaved for another 2 min, taken out and stirred, and then microwaved for a final 50 s and stirred again.

The mixed Group B and E ingredients were slowly and evenly added and stirred using a spoon-spatula into the cooked tomatoes and egg yolk mixture for 1 min before being continued stirred manually for 5 min. The flaxseed oil and the vitamin solutions were added at the end and manually stirred until well homogenized in the diet.

While the diet was still warm, it was poured into a 1500 mL squeeze bottle (diameter of hole dispenser lid: ±7 mm) (Ace Hardware, Jakarta, Indonesia). Next, 2 g portions of diet were poured into sterilized 30 mL plastic cups (Frontier Scientific, Newark, NJ, USA) and set under the laminar hood for 5 min to cool completely. The 30 mL cups with lids (dia: 3 cm, height: 3 cm) used for diet media were soaked using 70% ethanol for 12 h, dried out upside down above the trays, and placed under UV-light on a laminar airflow hood for one hour before having diet placed in them.

One sterilized egg on its Kimwipe square was placed on the prepared diet (as described above), with the egg facing upwards, before replacing the lids and labeling the cups with the date the diet was prepared and its formulation code.

#### 2.3.3. MM4

In this diet, fresh tomatoes were not used, being replaced with canned whole peeled tomatoes (CIRIO Pelati Peeled Plum Tomatoes, Savena, Italia). In addition, flaxseed oil was replaced with linseed oil. Additions to the diet included a vitamin mix fortification for Lepidoptera, chickpea and red kidney bean flour, white sugar, and an antimicrobial blend, with a stock solution made up as follows: 0.45 g sorbic acid dissolved in 9 mL of absolute ethanol in a 25 mL screwcap Erlenmeyer flask. Then, 0.075 g of tetracycline, and 0.685 mL of methyl benzoate were added and dissolved in 2.48 mL of sterile RO water. The solutions were shaken until completely dissolved and stored in a 25 mL screwcap Erlenmeyer flask. In addition, different amounts of raw egg yolk, ascorbic acid, brewer’s yeast, and phytagel were added. These are all detailed in Table 1. Diet mixing was performed in a similar fashion to that indicated for diet MM1, except that hand mixing, where possible, was replaced with mechanical mixing.

The dry ingredients (Group B: cellulose powder, lecithin granules, tapioca flour, brewer’s yeast, chickpea flour, and red kidney beans flour) were evenly mixed by hand using a stainless steel spoon in a 2000 mL stainless steel beaker and covered with aluminum foil.

The vitamins (Group C: Myo-inositol, choline chloride, ascorbic acid, vitamin mix fortification for Lepidoptera, and sugar) were dissolved in 50 mL of sterile RO water in a 100 mL screwcap Erlenmeyer flask at ambient temperature.

Phytagel (Group E) was weighed into a 1000 mL Erlenmeyer flask which had been filled with 450 mL of sterile RO water and immediately hand-shaken until there were no lumps. This gelling media (phytagel and RO water) and the dry ingredients were autoclaved at 115 °C for 20 min. Immediately after autoclaving, the dry Group B ingredients were added to the Phytagel mix, with a magnetic stirrer (length: 3 cm, PTFE, Mettler Toledo, Hamilton, New Zealand) and placed on a hotplate stirrer (IKA C-MAG HS 7, temp. 100 °C and Mot.1) to keep the phytagel mixture at boiling point.

The canned whole peeled plum tomatoes were blended in their juice to form a homogenous mix, using a Philips Blender (Viva collection, HR2116, Bogor, Indonesia). The surfaces of the chicken eggs (Group A) were washed with natural antibacterial liquid soap (before being sterilized in a 0.2% Sodium hypochlorite (NaOCl- chlorox) (Bayclin, Sudimoro, Indonesia), solution for 15 min, rinsed using sterile distilled water, and dried out under a laminar flow bench. The raw egg yolk, after being carefully separated from the egg white using an egg yolk separator, was placed in a 100 mL beaker and stirred until well-mixed before being added to the homogenized peeled whole plum tomato solution and further homogenized together using the blender. The homogenized tomato and egg yolk mixture was microwaved (Panasonic NN-ST342, 1270 W, Shanghai, China) on the high setting for 2 min, taken out and stirred, then microwaved for another 2 min, taken out and stirred, and then microwaved for a final 50 s and stirred again.

The mixed Group B and D ingredients were slowly added and evenly mixed into the cooked tomato and egg yolk mixture for 1 min before being blended using a hand-blender (Philips Promix 650 W, Amsterdam, Netherlands) for 5 min. The linseed oil, vitamin solutions, and antimicrobial blend were added at the end and blended until well homogenized in the diet.

While the diet was still warm, it was poured into a 1500 mL squeeze bottle (diameter of hole dispenser lid: ±7 mm) (Ace Hardware, Indonesia). Then, 2 g portions of diet were poured into sterilized 30 mL plastic cups (Frontier Scientific, Newark, NJ, USA) and set under the laminar hood for 5 min to cool completely. The 30 mL cups with lids (dia: 3 cm, height: 3 cm) used for the diet media were soaked in 70% ethanol for 12 h, dried out upside down above the trays, and placed under UV-light on a laminar airflow hood for one hour before having diet placed in them.

One sterilized egg on its Kimwipe square was placed on the prepared diet (as described above), with the egg facing upwards, before replacing the lids and labeling the cups with the date the diet was prepared and its formulation code.

The diet cups were placed on trays in incubators (Constant Climate Chamber KBF 240 E6, Binder, Tuttlingen, Germany) and kept at 27 °C and RH 70–90% until adult emergence.

All the glassware and steel implements used in preparing the diet were autoclaved at 115 °C for 20 min and dried at 60 °C overnight in an oven (Heratherm OMH180-S. Thermo Scientific, Langensbold, Germany) before use.

#### 2.3.4. pH and Water Activity

pH is an essential factor in an artificial diet. A too alkaline or too acidic pH can be detrimental to larval development and affect contamination [19,20]. Generally, insects require a slightly acidic pH range in their diets [19]. The pH of unripe mature pods from cocoa clone M01 cut in half was measured with a pH meter (Orion Versa Star Pro, Thermo Scientific, Waltham, MA, USA) coupled with a pH probe (Orion UltrapH/ATC triode, Thermo Scientific, Waltham, MA, USA) at 3 locations on the half pod (proximal, central, and distal sections) and averaged. The pH of the developed diets was compared with the cocoa pods’ pH range (Table 2).

Most plant-feeding insects require a 70–90% water content in their diet to complete their life processes [19]. In artificial diets, this water is bound by the gelling agents used in the diets. Free water in diets, measured as water activity (a_w_), is required by micro-organisms such as bacteria, mold, fungi, yeasts, etc., for growth. It is measured from 0 to 1, with 0 indicating fully free water, and 1 indicating fully bound water [21]. If diets have an a_w_ between 0.95 and 1, then there is very little free water available for microbial growth and thus diet contamination is minimized [21,22]. The a_w_ was measured using a water activity meter Aqualab 4TE (Meter Food, Pullman, WA, USA).

### 2.4. Conopomorpha Cramerella Development on the Artificial Diets

#### 2.4.1. Egg Hatching

One egg per cup was used in this experiment. An egg was considered fertilized and viable when the egg, which was observed under a stereomicroscope (Leica IC90 E, Singapore, Singapore) two days after transfer onto the diet, was found to be empty. At this stage, the egg walls are usually transparent, and a larval exit hole is visible. Eggs that did not hatch within 5 days after transfer onto the diet were considered dead. The duration between egg-laying and hatching was recorded, as were the numbers of hatched neonate larvae.

#### 2.4.2. Larval Developmental Success

The larval development was considered successful when the last larval instar exited from the diet and looked for a place to pupate, sometimes with and sometimes without spinning a cocoon. The larval exit and cocoon spinning behavior were checked daily for a week (seven days) after the egg hatched. Larvae that did not exit the diet within 30 days after the egg hatched were considered dead inside the diet. Most larvae usually emerged from the diet within 20 days. The duration between egg transfer into the diet and the last larval instar emerging from the diet was recorded, as were the numbers emerging.

#### 2.4.3. Cocoon Formation Success

In our experiments, the larvae exited the provided diet to locate a substrate for cocoon spinning and subsequent pupation. However, some larvae chose to pupate without spinning a cocoon, significantly reducing their chances of survival. Consequently, we compared the percentages of larvae successfully spinning a cocoon before pupation across the different diets tested.

#### 2.4.4. Pupation Success

Pupation success was measured by larvae successfully transforming into the pupal stage, with or without cocoon formation. Larvae that did not pupate within a week after exiting the diet died.

#### 2.4.5. Adult Emergence

Adult emergence was considered successful when the full adult emerged from the pupa, with or without malformation. It was considered unsuccessful if the adult did not emerge within two weeks after larval pupation started. The duration between the larva exiting the diet and the adult emergence from the pupa was recorded, as was the number and sex of the adults emerging.

#### 2.4.6. Adult Survival

Once they emerged, the adults were sexed and categorized into healthy or malformed adults. The duration between adult emergence and death was measured for each sex independently, as were the numbers dying. The body length and fresh weight of newly emerged adults were used as an indication of the overall fitness of the adults reared on the artificial diets in comparison with those reared on cocoa pods, as these factors are commonly monitored in laboratory insect populations as a measure of quality [23]. Newly emerged adults were collected the day after emerging from their pupal stage, and placed into an empty cup similar to the ones used for the diet experiments. The motionless adult’s fresh weight was measured using an analytical balance (Ohaus Adventurer AX 224, Ohaus corp., Oarsippany, NJ, USA), and LAZ (Leica Application Suite) Version 3.4.0 integrated with a CMOS microscope camera (Leica IC90 E, Leica, Singapore, Singapore) was used to measure body length.

#### 2.4.7. Sex Ratio

The sex ratio is usually a good indicator of a population’s health and the presence of adequate environmental and nutritional conditions. A sex ratio in lab-reared populations differing from that of wild populations usually underlines an existing problem in the rearing system or the diet composition. Examination of external genitalia [9] was used to determine adult sex. The hairy anal papillae of the ovipositor characterized the female genitalia, whereas a darker and wider caudal segment and the presence of a hair pencil confirmed the males. These observations were performed using an integrated CMOS microscope camera (Leica IC90 E, Leica, Singapore, Singapore).

### 2.5. Statistical Analysis

The diet comparison experiment consisted of thirty individual eggs per diet treatment (MM1 or MM4). Experiments were performed 17 times from November 2022 to April 2023. The success rates (%) and the duration for each development stage (days) were compared between treatments/diets using the Mann–Whitney U-test (Statistica 12; StatSoft, Tulsa, OK, USA). The fitness measurements (body size (mm) and weight (mg)) were compared between the MM4 diet and cacao pod-reared insects using the Mann–Whitney U-test (Statistica 12; StatSoft, Tulsa, OK, USA). Success rate and stage duration differences across generations were compared using the Kruskal–Wallis H test (Statistica 12; StatSoft, Tulsa, OK, USA). Results are given as mean ± SD, unless specified otherwise. Statistical significance was set at *p* < 0.05.

## 3. Results and Discussion

### 3.1. Diet Development

Since the publication of Awang et al. [18], significant advancements have been made in *C. cramerella* diet development. Between 2006 and 2019, the Malaysian Cocoa Board progressively upgraded the diet recipe to MM1, gradually eliminating all cocoa-related components from the list of ingredients. In our study, we present the success rates of this diet and the latest improved diet that has been developed thus far in Indonesia, jointly developed with the Malaysian Cocoa Board.

In addition, 200 g of the MM1 and MM4 artificial diets and dried cocoa pods were shipped to Eurofins Food Testing Singapore Pte. Ltd., (Singapore) for nutritional composition analysis. The nutritional compositions of both diets compared with the cocoa pod are shown in Table 2.

The preliminary analyses of the MM1 diet indicated that it may be too high in protein, while being too low in carbohydrate and vitamin C, compared with the cocoa pod host. To compensate for this, brewer’s yeast was reduced in the diet (from contributing 1.5% to 0.375% of the diet composition), and sucrose and ascorbic acid were increased in the diet to 0.33% and 0.085%, respectively. 

A further major change made to the MM1 diet was replacing fresh tomatoes with canned plum tomatoes. This eliminated much of the variability in the diet composition caused by differently aged tomatoes, different varieties used, and also the possible impact of pesticide residues on the fresh tomato surface on *C. cramerella* larvae feeding on the artificial diets. It has been clearly shown that different tomato varieties and ages of single varieties all have different pH, sugar, and nutrient contents [24,25], all of which impacted the nutritional quality of the early diets, including MM1. Similarly, the nutrient content of different canned tomato products also varies, as can be seen on the nutrition information printed on the product labels. It was found in trials using different tomato pastes, and products using different varieties of tomato that *C. cramerella* larvae developed best on diets containing CIRIO Pelati Peeled plum Tomatoes, canned in Savena, Italy (ABS, Pers. com.). In addition to stabilizing the level of certain nutrients, using canned tomatoes contributed to increasing the vitamin content in the new diet. However, the protein content remained high compared with the cocoa husk natural host plant. The egg yolk content was thus decreased from 10% to 5% of the diet volume. To reduce the fat content of the MM1 diet (also too high compared with cocoa husk), flaxseed oil was replaced with linseed oil, and the concentration was reduced from 4% to 1%.

In addition to the above, to further increase the vitamins and fiber content in the MM4 diet, chickpea flour (0.75%) and red kidney bean flour (0.75%) were added. The full diet compositions of the MM1 and MM4 diets are shown in Table 1. In all cases, the diet recipe modification resulted in reduced contamination, and improvements in quality and numbers of all insect stages produced from the diet, as indicated below.

This could be further improved as Table 2 shows that the MM4 diet still has much higher levels of fat, proteins, and amino acids than are contained in the cocoa husk, while it lacks total carbohydrates, dietary fiber, and sterols, especially stigmasterol and sitosterol. In terms of pH, however, the MM4 diet was comparable with the cocoa husk pH, being slightly acidic, as recommended in the literature for insect diets [19,22]. Similarly, the a_w_, being 0.98 for both diets, indicated that there was very little free water available for pathogen growth [21] in the diet. As long as good sanitation procedures are additionally followed in preparing and storing the diet [19,20], there is likely to be very low pathogen contamination in the prepared diets.

### 3.2. Conopomorpha Cramerella Development on the Artificial Diets

**Figure 1:**
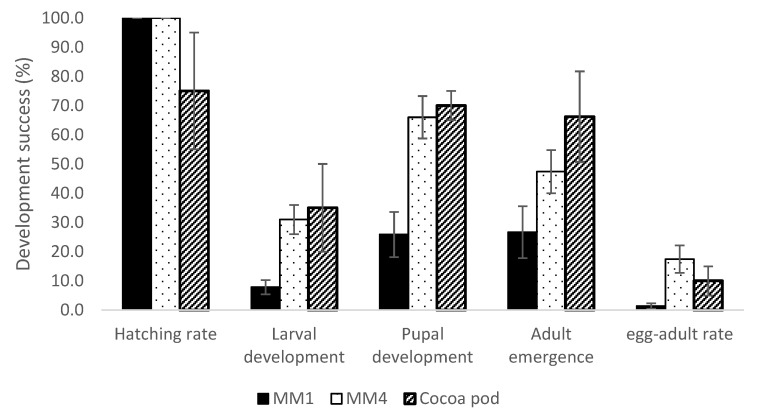
Hatching and development success rates (%, mean ± se) at each stage of the life cycle of *Conopomorpha cramerella* developing on the MM1 and MM4 diets (N = 17 repetitions, 30 eggs per treatment). The larval, pupal, and adult success rates of insects reared in cocoa pods were taken from [27,28,29], respectively.

Significant improvements were observed in all stages of the insect’s development. Egg hatching was consistently high (100%; Figure 1) for both diets, whereas only 75% hatching was reported from the natural host [26]. Larval development increased significantly (z = −3.583, *p* < 0.001) between the MM1 (below 10%) and MM4 (close to 40%) diets (Figure 1).

The improvement in diet formulation also led to significant enhancements in pupation success rates (z = 3.239, *p* = 0.001), with rates comparable to those achieved by cocoa-reared insects reported in the literature (Figure 1).

Similarly, the diet modifications from MM1 to MM4 increased the adult emergence success rates from the pupae stage (z = 0.374, *p* = 0.391) but without significant difference as only five adults emerged from the pupae developed on MM1, a rate similar to the adult emergence success rates from insects reared in natural conditions on cocoa pods reported in the literature (Figure 1).

**Figure 2:**
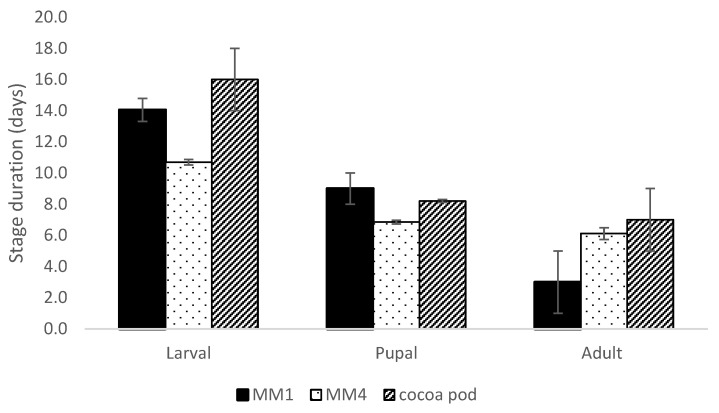
*Conopomorpha cramerella* larval and pupal development duration and adult longevity (mean ± se) developing on the two diets (MM1 and MM4) and cocoa pods. The larval, pupal, and adult durations of stages reared in cocoa pods were taken from [27,28,29], respectively.

The larval development period decreased from the first diet version (14 days) to the last (11 days) by 24% (z = 4.716, *p* < 0.0001), which was shorter than the 16 days for larval development within cocoa pods, as reported by [27] (Figure 2).

Adult longevity improved between MM1 and MM4 (z = −1.455, *p* = 0.169, only two adults emerging from MM1 survived) to become competitive with the adult lifespan of pod-reared insects (Figure 2).

The pupal period of insects reared on the MM4 diet decreased significantly (z = 2.210, *p* = 0.021) compared with those fed on MM1 (7 days vs. 9 days for MM4 and MM1, respectively). Wild pupae were reported at 8 days [29] (Figure 2).

**Figure 3:**
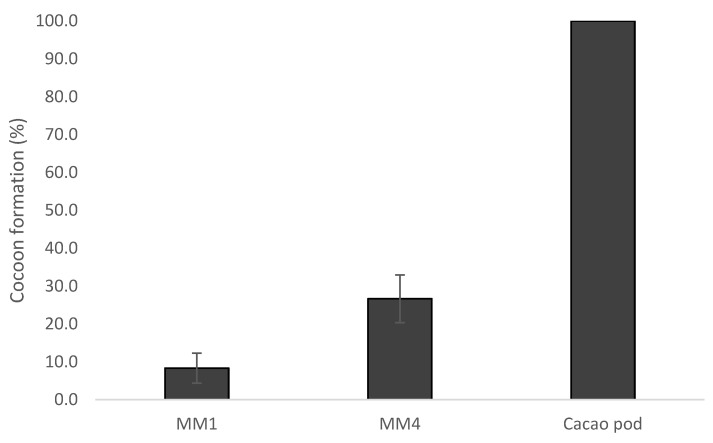
Success of spinning a cocoon (%, mean ± se) for *Conopomorpha cramerella* larvae reared on artificial diets (MM1 and MM4) and on cocoa pods.

Larvae emerging from diet MM4 were more successful than those from diet MM1 at building a cocoon to pupate in (z = −2.249, *p* = 0.026). However, the rate of cocoon formation was still less than half of that of larvae from cocoa pods [29] (Figure 3).

It has to be noted that *C. cramerella* can successfully pupate and emerge as an adult without a cocoon, but the absence of a cocoon probably explains the difference in adult emergence rates observed between our diet-reared insects and the cocoa pod-reared insects.

Overall, *C. cramerella* development success from eggs to adults improved significantly from diets MM1 to MM4 (z = −3.317, *p* = 0.002), reaching 17.4% (Figure 1). Only 3–16% of the larvae initially infesting cocoa pods managed to exit it successfully on different cocoa varieties in Indonesia [30]. These data for the host plant in Indonesia are consistent with other published work on cocoa pods in Malaysia [26]. In [26], 80% and 75% of the larvae succeeded in digging through the pre-sclerotic and sclerotic layers of the cocoa pod respectively, and after entering the pod, only 20% to 50% of the larvae successfully left the pod in search of a suitable pupation location.

The present study found that the overall development success of diet-reared insects from egg to adult in Indonesia was similar to the published development success of *C. cramerella* reared from cocoa pods [26,31] (Figure 1). This indicates that the current diet version shows relative promise in providing the nutrition needed for *C. cramerella* development as compared with those insects reared from cocoa pods. The laboratory-reared insects in Indonesia exhibited an overall lifespan of 27 days, which is similar to the lifespan of approximately 4 weeks (28 days) for wild individuals [24,25,26,31].

**Figure 4:**
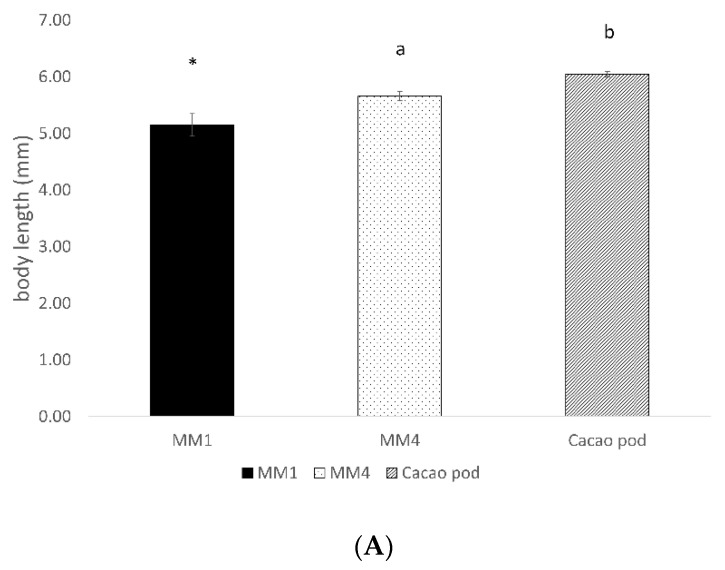

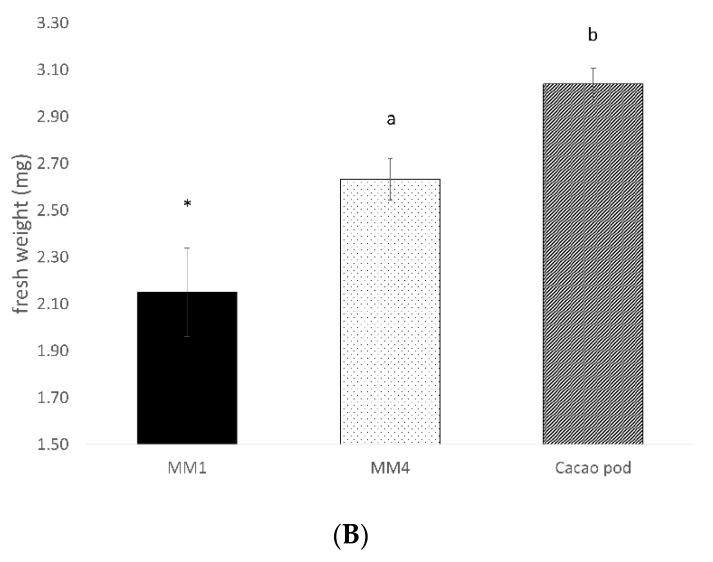
Adult body length (**A**) (mm, mean ± se) and fresh weight (**B**) (mg, mean ± se) of *Conopomorpha cramerella* fed on MM1 and MM4 diets, and cacao pods. Different letters indicate significant differences among treatments. * = number of emerging adults too low to be compared statistically.

The body length and fresh weight of newly emerged adults were used as an indication of the overall fitness of the adults reared on the two artificial diets in comparison with those reared on cocoa pods. Both measures of newly emerged *C. cramerella* adults varied significantly based on the food the larvae fed on, remaining lower for MM4 diet-reared insects compared with those feeding on cocoa pods (z = 3.506, *p* < 0.001 and z = 3.397, *p* < 0.001 for length and fresh weight, respectively) (Figure 4A,B).

The sex ratio of the lab-reared adults from the MM4 diet differed from that of the wild population, with a ratio of 1.3:1 recorded in the present study compared with the ratio of 1:1 recorded from those emerging from cocoa pods collected in the field [31].

**Figure 5:**
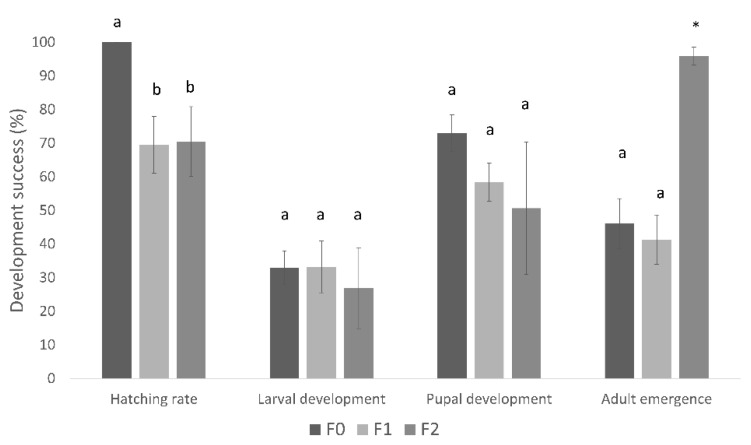
Hatching and development success rates (%, mean ± se) at each stage of the life cycle of *Conopomorpha cramerella* developing on the MM4 diet over three generations. Different letters indicate significant differences among treatments at each developmental stage. * = number of emerging adults too low to be compared statistically.

The primary objective of this study was to formulate an artificial diet for *C. cramerella* that enables the insect to develop from the egg through to the adult stage successfully and in numbers. The next phase would be to develop a laboratory colony solely from the adults reared on the artificial diet, to overcome the “bottleneck effect” [19,30] when colonizing insects collected from the wild into very different conditions in the laboratory. However, pilot studies using the MM4 diet have reared three generations of diet-reared insects, confirming the fertility of the F0 (Field collected) and F1 generations (with sex ratios of 1.3:1 and 1.1:1, respectively), but there were insufficient F2 adults produced to determine their sex ratio. The hatching success rate of eggs derived from diet-reared insects in both the F1 and F2 generations was found to be comparable (z = 0.549, *p* = 0.595); however, it was significantly lower when compared with the hatching rate of eggs from wild individuals (F0) (H(2, N = 37) = 24.137, *p* < 0.001) (Figure 5).

**Figure 6:**
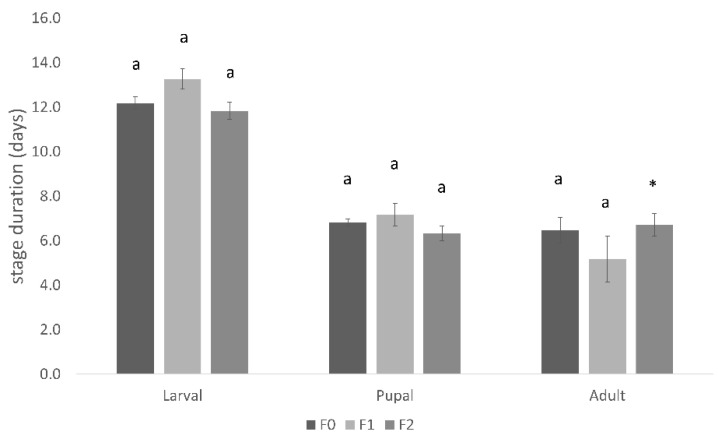
*Conopomorpha cramerella* larval and pupal development duration and adult longevity (mean ± se) developing on the MM4 diet in successive generations. Different letters indicate significant differences among treatments at each developmental stage. * = number of emerging adults too low to be compared statistically.

The rates of larval and pupal development success rates were similar across all three generations (H(2, N = 35) = 0.351, *p* = 0.839 and H(2, N = 28) = 3.731, *p* = 0.155, respectively). No differences were recorded for the larval and pupal durations, nor were there any differences in adult lifespan between generations (H(2, N = 212) = 4.642, *p* = 0.098; H(2, N = 70) = 2.425, *p* = 0.298, and z = 1.401, *p* = 0.161 for larva, pupa, and adult stages, respectively) (Figure 6). In terms of adult emergence success, the F2 generation exhibited a seemingly higher rate; however, the number of adult individuals emerging in this generation was insufficient to be compared statistically (Figure 5). The F2 was fertile.

In conclusion, the MM4 diet has shown a significant improvement in the successful rearing of *C. cramerella* from egg to adult, in terms of both the development of the different life stages and the fitness parameters when compared with the previous diet. These parameters were similar to those recorded for life stages feeding on cocoa pods and their wild hosts. By refining the diet constituents as indicated in the nutrition analyses presented in Table 2 and discussed above, it is expected the diet will become even more suitable for successfully rearing and establishing laboratory colonies of *C. cramerella.* These developments will contribute to future research and population mitigation studies, such as examining the potential use of SIT through radiation biology and adult marking trials, which will contribute to overcoming the significant challenge to cocoa production posed by this insect in Southeast Asia.

## Figures and Tables

**Table 1:** The composition of the original (MM1) and current (MM4) artificial diets used for laboratory rearing of *Conopomorpha cramerella*. Ingredients are categorized into groups A–G based on their types: A: fresh ingredients, B: dry ingredients, C: vitamins and minerals, D: lipids, E: gelling agent, F: ascorbic acid, G: decontaminating solution.

Group	Ingredients	Source	Unit	Diet MM1	Diet MM4
A	Fresh tomato	Tomoni Local Fresh Market, Indonesia	g (%)	500 (40.0)	-
	Canned tomato	CIRIO 1856, Pelati, Peeled plum Tomatoes, Savena, Italy	g (%)	-	500 (42.8)
	Fresh egg yolk	Tomoni Local Fresh Market, Indonesia	g (%)	100 (8.0)	50 (4.3)
B	Tapioca flour	Papioka, Real Organic Tapioca (Gluten Free), Pati, Indonesia	g (%)	15 (1.2)	15 (1.3)
	Lecithin granules	NOW, Non-GMO Lecithin Granules, IL, USA	g (%)	15 (1.2)	15 (1.3)
	Cellulose	Sigma Aldrich, Beijing, China	g (%)	30 (2.4)	30 (2.6)
	Brewer’s Yeast	Sigma Aldrich, Sao Paulo, Brazil.	g (%)	15 (1.2)	3.75 (0.3)
	Chickpea flour	Bob’s Red Mill, Stone Ground, Non-GMO Chickpea flour, Oregon, USA	g (%)	-	7.5 (0.6)
	Red kidney bean flour	Lingkar Organik, Tepung Kacang Merah Organik, Yogyakarta, Indonesia	g (%)	-	7.5 (0.6)
C	Myo-inositol	Duchefa Biochemie, BH Haarlem, The Netherlands	g (%)	1 (0.1)	1 (0.1)
	Choline chloride	Sigma Aldrich, Beijing, China	g (%)	1 (0.1)	1 (0.1)
	Vitamin fortification mix for Lepidoptera	Frontier Scientific, Newark, DE, USA	g (%)	-	0.85 (0.1)
	White Sugar	Gulaku, Lampung, Indonesia	g (%)	-	3.3 (0.3)
	Sterile RO water	Onelab Waterone, Deionized water, double RO, EDI, ultrafilter, UV. Jawa Timur, Indonesia water,	mL (%)	20 (1.6)	50 (4.3)
D	Flaxseed oil	Nature’s Gift, Omega Gold, Sabah, Malaysia	mL (%)	40 (3.2)	
	Linseed oil	Winsor and Newton, London, UK	mL (%)		10 (0.9)
E	Phytagel	Sigma Aldrich, Beijing, China	g (%)	30 (2.4)	20 (1.7)
	Sterile RO water	As above	mL (%)	380 (30.4)	450 (38.5)
F	Ascorbic acid	Swanson, Pure Vitamin C powder, ND, USA.	g (%)	1.7 (0.1)	0.85 (0.1)
	Sterile RO water	As above	mL (%)	100 (8.0)	-
G	Antimicrobial blend *		mL (%)	-	2.5 (0.2)
	Total Diet Volume		mL (%)	1248.7 (100)	1168.25 (100)

* = 11.25 mL stock solution (0.45 g sorbic acid (Sigma Aldrich, Beijing, China) dissolved in 9 mL of absolute ethanol with 0.075 g of tetracycline (Sigma Aldrich, Beijing, China), 0.685 mL of methyl benzoate (Merck, Darmstadt, Germany), dissolved in 2.48 mL of sterile RO water. Solutions were shaken until completely dissolved and stored in a 50 mL screwcap glass bottle in the fridge).

**Table 2:** Comparison of the nutritional composition of the MM1 and MM4 diets prepared in Indonesia with mature cocoa pods. The data are given as a percentage of dry weight. (* = data under detection level).

	MM1	MM4	Cocoa
pH	5.7	4.8	5.04
Water content (%)	80.2	84.5	88.3
Water activity	0.988	0.985	
Ash (%)	3.9	4.3	5.8
Fat (%)	35.9	16.8	0.9
Protein	14.1	12.6	8.2
Total Carbohydrates	46.5	66.5	84.6
Total Dietary Fiber		32.7	57.4
Total Sterols	83.8	66.5	78.5
Saturated Fatty Acids	6.1	3.6	*
Total Cis Unsaturated Fatty Acids	24.9	12.1	*
Monounsaturated Fatty Acids	9.0	5.0	*
Polyunsaturated Fatty Acids	15.9	7.0	*
Trans Fatty Acids	0.0	0.0	*
Omega 3 Fatty Acids	10.7	2.6	*
Omega 6 Fatty Acids	5.9	4.7	*
Omega 9 Fatty Acids	8.4	4.7	*
Total Fatty Acids	32.4	16.3	*
16:0 Palmitic	4.7	2.9	*
16:1 Palmitoleic	0.5	0.2	*
18:0 Stearic g	1.7	0.9	*
9c 18:1 Oleic	8.4	4.7	*
18:2 Linoleic	5.7	4.6	*
18:3 Gamma Linolenic	0.0	0.0	*
18:3 Alpha Linolenic	10.7	2.6	*
20:4 Arachidonic (n6)	0.2	0.2	*
22:6 Docosahexaenoic	0.0	0.0	*
Total 18:1 trans	0.0	0.0	*
Total 18:1 cis	0.0	0.0	*
Amino Acids			
Aspartic Acid	1.27	1.39	0.76
Threonine	0.58	0.48	0.22
Serine	0.83	0.66	0.25
Glutamic Acid	2.09	2.52	0.58
Proline	0.48	0.39	0.30
Glycine	0.45	0.36	0.24
Alanine	0.68	0.54	0.31
Valine	0.68	0.52	0.30
Isoleucine	0.63	0.47	0.23
Leucine	0.97	0.77	0.38
Tyrosine	0.50	0.39	0.21
Phenylalanine	0.55	0.47	0.23
Lysine	0.87	0.64	0.35
Histidine	0.28	0.25	0.14
Arginine	0.75	0.79	0.23
Cystine	0.19	0.13	*
Methionine	0.33	0.22	0.09
Tryptophan	0.15	0.12	*
Cholesterol	0.58	0.39	*
Campesterol	0.02	0.01	*
Stigmasterol	0.01	0.01	0.02
Beta Sitosterol	0.03	0.02	0.06
Brassicasterol	0.00	0.00	*
Other Sterols/Stanols	0.03	0.03	*

## Data Availability

The data supporting this study’s findings are available from the corresponding author, Jerome Niogret, upon reasonable request.
